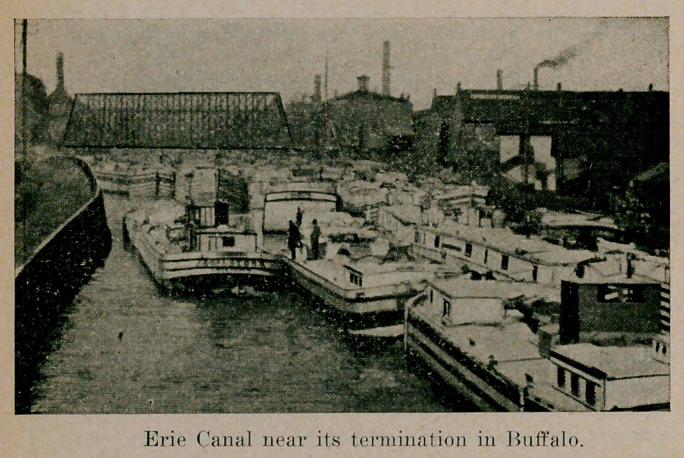# Pellagra—A Case Reported

**Published:** 1915-04

**Authors:** Joseph Spangenthal

**Affiliations:** Dermatologist to the Good Samaritan Dispensary and Emergency Hospital, Buffalo, N. Y.; 595 Lafayette Avenue


### Pellagra—A Case Reported.

By JOSEPH SPANGENTHAL, M. D.

Dermatologist to the Good Samaritan Dispensary and Emergency Hospital, Buffalo, N. Y.

Pellagra is a disease that has been recently recognized in this country, and is becoming more and more prevalent in the southern states.

Sporadically, it has been discovered in almost every state in the Union. On account of its rare occurrence in this section of the Country, we think it of sufficient importance to place on record our case, together with several others that have been observed in Buffalo and vicinity.

The patient, L. K. male, aged 35 years, tall and slender, is of American birth. He came from a Pennsylvanian town of 2000 inhabitants.

FAMILY HISTORY:

The patient's maternal grandfather died at the age of 94. The maternal grandmother died at the age of 86. The cause of their death is unknown. The history of the father's parents is not obtainable. His father died at the age of 76; the cause of death is not known. His mother is still living at the age of 70; her health has always been very poor on account of chronic rheumatism. Three brothers and three sisters are enjoying good health. There is no history of neurosis or insanity in the family, including aunts, uncles, and cousins.

PAST PERSONAL HISTORY:

During childhood, patient had measles, mumps, whooping cough and scarletina. He has never had malaria or syphilis. His health has always been exceptionally good. Twelve years ago, he thought he had contracted syphilis (syphiliphobia), and this caused him to use alcohol immoderately.

HISTORY OF THE DISEASE:

In August, 1913, the patient came to Buffalo for consultation. He appeared quite despondent, but there were no delusions; he wished to consult us on account of a skin eruption, which will be described presently. His physical condition was poor, having lost considerably in weight.

URINALYSIS:

Specific gravity, 1020; urea 2½%. Sugar and albumin absent.

DIGESTIVE APPARATUS:

Appetite was poor; bowels were regular; (there was no indication of diarrhea at this time).

VASCULAR SYSTEM:

There was nothing abnormal to be heard upon cardiac examination; the pulse was regular and the rate varied between 80 and 90. Blood pressure was 140.

BLOOD EXAMINATION:

Haemaglobin 88%; leucocytes 11050; polynuclears 72%; small lymphocytes 23%; large lymphocytes 1%; transitionals 2%; basophiles 1%; eosinophiles 1%.

WASSERMAN BLOOD TEST:

Negative.

NEUROLOGICAL EXAMINATION:

Mental condition: Intelligent; memory good; worries somewhat about his condition.

Pupils are slightly unequal, right being larger than left; both react to light and distance.

Motor function: No incoordination nor ataxia; gait normal.

Sensory function: Negative.

Reflexes: The patellar reflex is normal; plantar reflex is present; there is no Babinski.

SKIN:

The back of both hands was covered with blebs which had ruptured, and resembled a burn of the second degree; the skin was macerated and was covered with pus and blood. The backs of the fingers were deeply pigmented; the lesions extended upon the forearms for several inches, forming a line of demarcation, which showed the characteristic notching .

A small dry pigmented patch was present on one wrist; a linear pigmented lesion was seen on the back of the forearm reaching to the elbow.

The palmar surface of both hands and fingers was clear. The epidermis of the face was dry as in Ichthyosis, and pigmented; the lips were dry and parched; the tongue and buccal mucous membrane were deep red, glazed, and were covered with whitish patches and pustules.

TREATMENT:

The treatment of pellagra with Salvarsan has engaged the attention of the medical profession within recent times. In an article by Willis E. Stern, Decatur, Ala., one case is reported of a mulatto aged 37, who was given an intravenous injection of Salvarsan; all manifestations of the disease cleared within two days, and the skin became normal in appearance within 15 days.

W. J. Cranston, Midgeville, Ga., reports 10 cases, of which 2 left the hospital clinically cured; 3 had a relapse and were treated a second time; 1 was unimproved, and 4 died.

To this list we wish to record our case, who had received 2 intravenous injections of Salvarsan.

On August 14th 0.4 Salvarsan was given intravenously. Excepting a slight headache, the patient reacted splendidly. August 18th the bandages were removed from both hands, and the change was as remarkable as we could have anticipated after a similar treatment for syphilis. New epidermis had already formed upon the raw surfaces; the old epidermis was desquamating as in Scarletina. The same satisfactory results were observed in the mouth; the pustules had all disappeared, and the mucous membrane of the tongue, pharanx, and buccal surface, was losing its inflammatory aspect.

On August 23rd, another intravenous injection was given (0.5).

The patient seemed highly elated upon his improved condition, both physical and mental.

September 17th, the patient returned to Buffalo. The epidermis of hands, feet and face had become very dry, rough, and deeply pigmented. His appetite was very poor, and he was suffering with diarrhoea of a severe type. It was evident that the disease, although temporarily benefited by the Salvarsan, was recurring. Opium was given to check the diarrhoea, and Quinia Hydrobromide empirically. Several weeks later his health was much improved, having gained 15 pounds in weight, and the skin had almost a normal appearance.

Through the courtesy of Dr. W. A. Ostrander, we are enabled to report the history of a second case of Pellegra, occurring in McKean county.

The patient of whom the doctor writes, lived on a farm, in the same county as does our patient. She was 33 years of age, born of Swedish parents, and has always lived in the same county. She was married about 14 years ago, and had two healthy children. During childhood, the patient suffered from the ordinary children's diseases. In May 1911, she consulted her physician on account of a rash on her hands, which at that time was diagnosed Poison Ivy Dermatitis. She stated that she handled Phosphate in planting potatoes. The rash extended to her wrists and forearms, and a few spots appeared on her face. Later the palms of both hands became covered with thickened epidermis, so that she could flex her fingers with great difficulty. In the spring of 1912, her fingers appeared ankylosed, and were very painful. The backs of her hands were covered with a dermatitis characteristic of Pellagra.

Gastro-intestinal derangement then followed. Under the administration of the Iodides, she seemed to improve, but later relapsed into her former condition. Ultimately she drifted into Melancholia with delusions of persecution. Her gums would bleed and had the appearance of scurvy.

She refused to eat and talk for days, and had spells of weeping. In May 1912 the patient died. The Physician also writes that in the Fall of 1910, the corn in McKean county was not good; that the ears were decayed on the tops; but that the bottom was eaten.

To these two isolated cases we wish to offer the salient features of two more cases; one from Buffalo, the other from Hornell, N. Y.

Buffalo case: (from an article by G. W. Wende, Buffalo Medical Journal; May, 1911.)

This, the first indigenous case reported in New York State, appeared in a woman aged 33 years. She had received during her whole lifetime good nourishing food in abundance. Corn and maize never constituted much of her diet. She had never been outside of the county, excepting on her wedding trip ten years previously, when she spent ten days in New York City.

Referring to this article, we note that in one year alone, Buffalo handled 20 million bushels of corn, which came via the lakes. This had become overheated in the vessels en route. After drying by fan system, the corn was reshipped to New England markets for consumption.

The damaged corn which was not accepted by the consignee, was kiln dried, ground and made into chicken feed. In the states in which this corn was sold, there have been but three cases of Pellagra reported. The states in which Pellagra appears endemically, receive shipment from the same source as does Buffalo.

Hornell case: The patient's parents were born in Steuben county, and had spent their lives there. The patient aged 42, was born in Steuben County, and was never outside of the county. She was brought up in poverty, and gives the history of having eaten very little of cornmeal.

In considering the principal features in the histories of the above cases, we are led to the conclusion that the previously considered corn or maize theory should not be acceptable for the following reasons:

1. Very little corn or maize entered into the diet of the Buffalo and Hornell cases.

2. If spoilt corn were the offending factor, one would expect a history of Pellagra in endemic form in McKean County, during the year that the corn was rotted; whereas only two cases were reported.

3. The states where Pellagra occurs endemically, and those where it is of rare occurrence, including the city of Buffalo, receive their corn supply from the same source.

In conclusion, as a suggestion to the etiology of Pellagra, it seems that Malaria would offer an excellent example. Both Malaria and Pellagra are commonly found in the Southern states and in some of the Western States; yet they are rarely encountered in the North. We have learned and proven the etiology of Malaria to be a transmitted organism, carried by a certain specie of mosquito. May we not hope to discover a parallel cause for Pellagra?

595 Lafayette Avenue.

## Figures and Tables

**Figure f1:**
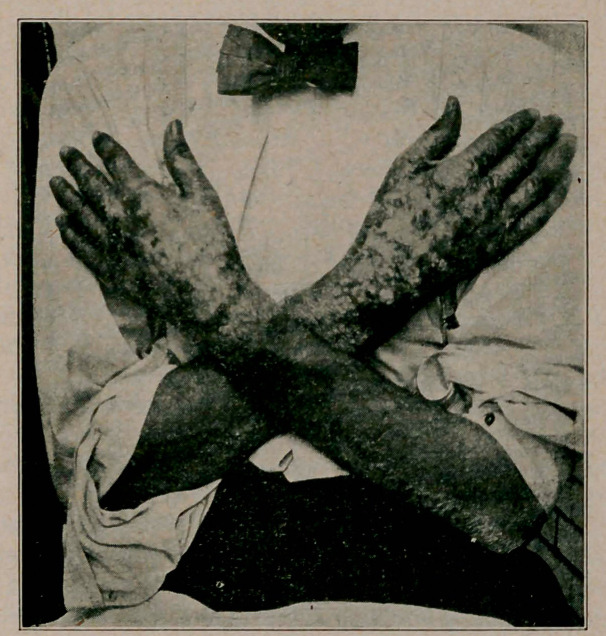


**Figure f2:**